# Spillover effects of organizational support for patient and workplace safety on safety outcomes: The mediating role of safety compliance

**DOI:** 10.1111/jan.16222

**Published:** 2024-05-09

**Authors:** Ja Kyung Seo, Seung Eun Lee

**Affiliations:** ^1^ Department of Psychology Psychological Science Innovation Institute, Yonsei University Seoul South Korea; ^2^ Mo‐Im Kim Nursing Research Institute, College of Nursing Yonsei University Seoul South Korea

**Keywords:** organizational support, patient safety, safety compliance, safety outcome, workplace safety

## Abstract

**Aim(s):**

To investigate spillover effects of organizational support for patient and workplace safety on safety outcomes and to examine the mediating role of safety compliance in these relationships.

**Design:**

A cross‐sectional, correlational survey design.

**Methods:**

This study analysed data from 1255 nurses in 34 Korean hospitals. A structured questionnaire was used including items from the Hospital Survey on Patient Safety Culture and Safety Compliance scales. Data were collected between February and June 2022. We employed structural equation modelling (SEM) for analysis with a significance level set at 0.05.

**Results:**

Organizational support for patient and workplace safety showed direct impacts on patient and workplace safety outcomes. Findings supported our hypotheses regarding spillover effects, as organizational support for patient safety was related to enhanced workplace safety and organizational support for workplace safety was associated with improved patient safety. SEM analysis showed safety compliance's mediating role. When the distribution of serial indirect effects was examined, three out of eight indirect pathways were statistically significant.

**Conclusion:**

Improving organizational support for patient safety can lead to better workplace safety outcome, and enhancing support for workplace safety can result in better patient safety outcome. Given this mutually beneficial relationship, healthcare organizations should simultaneously promote safety in both areas rather than focusing on just one.

**Implications for the Profession and/or Patient Care:**

Study results highlight the need to recognize the interconnected nature of patient and workplace safety in order to achieve better overall safety outcomes.

**Impact:**

This study shows that organizational safety efforts for patients and workers are interconnected and mutually beneficial. The study's results have both theoretical and practical implications in demonstrating that organizational support for both patient and workplace safety plays a strong role in promoting nurses' safety compliance and improving overall safety outcomes.

**Reporting Method:**

STROBE checklist.

**Patient Contribution:**

No patient or public contribution.

## 1 INTRODUCTION

Ensuring a safe healthcare environment for both patients and workers has become more critical than ever, especially in light of the COVID‐19 pandemic. This situation has emphasized the hazardous and unpredictable nature of healthcare working environments (Aljabri et al., 2020; Organisation for Economic Co‐operation and Development, 2021), underscoring the importance of addressing both patient and workplace safety in a cohesive manner. Patient safety refers to ensuring that patients are not harmed due to preventable causes and that the risk of unnecessary harm related to healthcare is reduced to an acceptable level (World Health Organization [WHO], 2023). Workplace safety is characterized as a level of work system quality that minimizes the probability of causing harm, whether instantly or over time, to individuals while performing work (Beus et al., 2016). Although distinct, patient safety and workplace safety share the common thread of prioritizing safety and reducing preventable harm within healthcare settings (Flin, 2007; World Health Organization, 2023).

The positive impact of organizational support on patient safety and workplace safety has been studied in each of these areas. For example, targeted organizational support towards patient safety was associated with improved patient outcomes (Stimpfel et al., 2019). Similarly, a beneficial link was identified between organizational efforts for workplace safety and enhanced safety outcomes for employees (Vu et al., 2022). Nevertheless, Flin (2007) emphasized that the way in which management and supervisors prioritize safety shapes the overall safety climate within an organization, and this, in turn, influences the deliberate safety behaviours of workers, impacting both patient and worker safety outcomes. Such insights indicate that fostering patient and workplace safety can play a crucial role in risk management, safeguarding both healthcare recipients and providers. Despite a possible connection between patient and worker safety (American Association of Colleges of Nursing, n.d.), such an interconnection has not been sufficiently explored (Kim et al., 2023), marking a significant oversight in the existing literature. Addressing this gap, our research aims to investigate how organizational support for safety can simultaneously enhance patient and workplace safety in healthcare organizations.

## 2 BACKGROUND

Previous studies have shown that safety climate is a key factor influencing safety outcomes in that it reduces safety incidents and promotes nurses' patient safety behaviours (Brunetto et al., 2021; Neal & Griffin, 2006; Seo & Lee, 2022). The perception of a safe climate is influenced by the degree of organizational emphasis on safety, which is often demonstrated through the actions of management and supervisors (Mashi et al., 2020). Flin also (2007) argued that organizational safety climate could be shaped by the combination of management and supervisor support, which could impact safety outcomes for both patients and workers. Bisbey et al. (2021) have emphasized that organizational enabling factors—such as management that supports safety policies and resources and leaders who prioritize safety—are prerequisites for establishing safe environments and behaviours, which ultimately result in improved safety outcomes. Indeed, positive impacts of organizational support on patient safety and workplace safety have been studied in each of these areas. For example, a positive relationship was found between organizational support for patient safety and improved safety outcomes for patients (e.g., Labrague et al., 2022). Similarly, organizational support aimed at workplace safety was associated with favourable outcomes such as reduced workplace injuries (e.g., Kao et al., 2021). This body of evidence underscores the significance of organizational support in achieving better safety outcomes. Thus, we proposed the following hypotheses.

Organizational support for patient safety would have a positive effect on patient safety.

Organizational support for workplace safety would have a positive effect on workplace safety.

Employees tend to exhibit less safe behaviours when they perceive that management places less emphasis on safety‐related policies, strategies, procedures and activities (Seo & Lee, 2022; Vinodkumar & Bhasi, 2010; Vu et al., 2022). In healthcare, this tendency could be particularly critical. Nurses are required to perform in‐role safety activities, such as adhering to standard safety rules and wearing personal protective equipment (PPE), which are referred to as ‘safety compliance’ (Gracia et al., 2020). Safety compliance is known to be strongly influenced by safety climate, including perceived safety‐related organizational support and management's safety priority (Bayram et al., 2021; Mirza et al., 2019). Similarly, employees are more likely to comply with safety rules and procedures if they perceive fairness and consistency in their supervisor's safety‐related actions and the organization's support for safety (Lee et al., 2019). Given this evidence, it is reasonable to assume that when management and supervisors actively promote safety, nurses would be more likely to engage in safety compliance. Thus, the following hypotheses are put forward.

Organizational support for patient safety would be positively related to patient safety compliance.

Organizational support for workplace safety would be positively related to workplace safety compliance.

Flin (2007) suggested that unsafe worker behaviours, such as rule‐breaking and risk‐taking, are key predictors of adverse safety outcomes not only for patients but also for workers themselves. In fact, the link between safety compliance and safety outcomes in healthcare is well documented. For example, safety compliance was negatively related to undesired injuries, accidents and near misses (Hu et al., 2021), and compliance with safety actions was positively associated with quality of care (Labrague et al., 2021). Accordingly, we proposed the following hypotheses.

Patient safety compliance would be positively related to patient safety.

Workplace safety compliance would be positively related to workplace safety.

Prior research has hinted at the existence of these “spillover” effects, a mechanism often observed in psychological research to examine the impact of experiences in one domain on experiences in the other, but related, domain (Bakker et al., 2009). Spratt et al. (2012), for instance, asserted that a focus on workplace safety would benefit both patients and healthcare workers. Another study found that continuous quality improvement initiatives aimed at improving organizational systems and processes were associated with higher process quality and safer patient care (McFadden et al., 2015). A more recent study also found that improving the working conditions of hospital staff not only enhanced their perceived working conditions but also boosted the overall quality of care (Sturm et al., 2019). In addition, drawing on Flin's (2007) model of safety climate and injury outcomes, it is likely that the influence of organizational support for patient safety and workplace safety on corresponding safety outcomes is manifested in two distinct forms of safety compliance: patient safety compliance and workplace safety compliance. Yet evidence is lacking that patient safety compliance can improve workplace safety and that workplace safety compliance can improve patient safety. Thus, we proposed the following hypotheses.

The positive effects of organizational support for patient safety and workplace safety on safety outcomes would be mediated by patient safety compliance and workplace safety compliance.

## 3 THE STUDY

**FIGURE 1:**
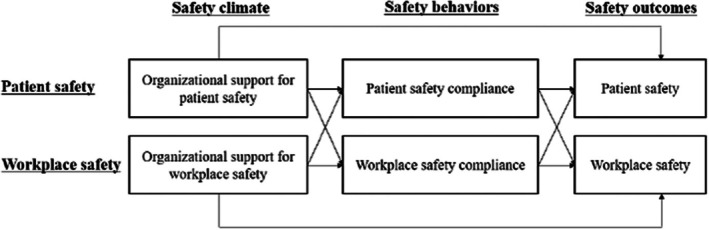
Hypothesized model.

This study aimed to investigate the spillover effects of organizational support for patient safety and workplace safety on safety outcomes and to examine the role of safety compliance in these relationships. Specifically, we seek to understand whether organizational support in one domain (patient safety or workplace safety) not only enhances safety compliance and outcomes within that domain but also exerts a positive spillover effect on the other domain. Through this investigation, we aimed to elucidate the interconnected nature of patient safety and workplace safety. This research is underpinned by the theoretical model (Flin, 2007) that a safety climate, fostered by organizational support, can lead to improved safety compliance among nurses, thereby enhancing both patient safety and workplace safety outcomes. Figure 1 illustrates the proposed model.

## 4 METHODS

### 4.1 Design, setting and sample

In this cross‐sectional, correlational study, we analysed data obtained from a larger research project that focused on investigating workplace safety and patient safety in acute care hospitals. Our study employed data from 1255 nurses working in 34 Korean hospitals. Given the numerous variables associated with hospitals' structures and functions, random sampling was not feasible. Instead, to maximize sample representativeness, we conveniently selected hospitals according to their locations across seven metropolitan areas and nine provinces in Korea. The nurse data were collected from various general and specialized units between February and June 2022. Eligibility criteria required that participants have a minimum of 6 months of clinical experience and be directly involved in patient care. Nurses holding managerial roles were not considered due to previously observed discrepancies between staff nurses' and managers' perceptions of work environments and care quality (Gormley, 2011; Shuman et al., 2023). More detailed information about sampling is presented elsewhere (Lee et al., 2024). A sample size of 1255 was deemed adequate to attain a power of 0.8 for detecting small effects in structural equation modelling (SEM) analysis with the use of bias‐corrected bootstrapping (Fritz & MacKinnon, 2007).

### 4.2 Measures

#### 4.2.1 Predictor variables

One predictor variable, *organizational support for patient safety*, was measured using items from the Hospital Management Support for Patient Safety subscale (2 items) and from the Supervisor, Manager or Clinical Leader Support for Patient Safety subscale (2 items) of the Korean version of the Hospital Survey on Patient Safety Culture 2.0 (HSOPSC 2.0) (Lee & Dahinten, 2021). Each of the subscales includes one negatively worded item, which we excluded to address criticisms of negatively worded items that can cause confusion among survey respondents and to improve the clarity and consistency of the responses (Ahmed et al., 2023; Palmieri et al., 2020). A sample item is ‘Hospital management provides adequate resources to improve patient safety’. The second predictor variable, *organizational support for workplace safety*, was assessed using items from the Hospital Management Support for Workplace Safety subscale (3 items) and the Supervisor, Manager or Clinical Leader Support for Workplace Safety subscale (3 items) of the HSOPSC Workplace Safety Measures (Agency for Healthcare Research and Quality [AHRQ], 2023). An example item is ‘The actions of hospital management show that the safety of providers and staff is a top priority’. For both measures, responses were rated on a 5‐point Likert scale ranging from 1 (*strongly disagree*) to 5 (*strongly agree*). The average score for each scale was calculated, and a higher score indicated a higher level of each construct. For the study sample, the Cronbach's alpha for organizational support for patient safety was 0.82, and the value for organizational support for workplace safety was 0.91.

#### 4.2.2 Mediator variables

One mediator variable, *patient safety compliance*, was measured using a three‐item scale that had shown good psychometric properties with the Korean nurse population (Seo & Lee, 2022). An example item is ‘I use the correct patient safety‐related procedures for carrying out my job’. The second mediator variable, *workplace safety compliance*, was assessed using a three‐item measure that showed construct validity and reliability (Neal & Griffin, 2006). A sample item is ‘I use all the necessary safety equipment to do my job’. For both instruments, responses were rated on a 5‐point Likert scale ranging from 1 (*strongly disagree*) to 5 (*strongly agree*). The mean score for each measure was calculated, and a higher score indicated a higher level of each construct. For the study sample, the Cronbach's alpha for patient safety compliance was 0.84, and the value for workplace safety compliance was 0.84.

#### 4.2.3 Outcome variables

One outcome variable, *patient safety*, was measured using a single item from the Korean version of the HSOPSC 2.0. This item asked nurses to rate overall patient safety in their work units (Lee & Dahinten, 2021). The second outcome variable, *workplace safety*, was assessed using a single item from the HSOPSC Workplace Safety Measures (AHRQ, Agency for Healthcare Research and Quality, 2023), which asked nurses to rate overall workplace safety in their work areas. For both measures, responses were rated on a 5‐point rating scale ranging from 1 (*poor*) to 5 (*excellent*). Previous research had demonstrated that the two single‐item measures were psychometrically sound (Lee & Dahinten, 2021; Zebrak et al., 2022).

#### 4.2.4 Demographic information

Demographic data collected from nurses included age, educational level, employment status, gender, hospital tenure, unit tenure and work unit.

### 4.3 Data analysis

Before proceeding with hypothesis testing, we performed initial analyses using SPSS version 29.0. Descriptive statistics (means, standard deviations and percentages) were calculated to represent the demographic characteristics of the study participants. Pearson's bivariate correlations were computed to examine the relationships between key study variables. Additionally, variance inflation factor, skewness and kurtosis values were examined to assess whether the data violated multicollinearity or normality assumptions. Next, confirmatory factor analysis (CFA) and SEM were conducted using Mplus version 7.0 to evaluate the measurement model and hypothesized model. Before conducting SEM, CFA was performed to confirm the construct validity of the latent variables and to ascertain whether the data fit the hypothesized measurement model. The robust maximum likelihood method was applied for model estimation. The adequacy of model fit was evaluated based on the following goodness‐of‐fit indices: the comparative fit index (CFI) > 0.90, Tucker‐Lewis index (TLI) > 0.90, root mean square error of approximation (RMSEA) < 0.08, and standardized root mean square residual (SRMR) < 0.08 (Browne & Cudeck, 1992).

In our SEM analyses, participants' age, hospital tenure, unit tenure, and educational level were controlled for, as they were significantly correlated with patient safety compliance, workplace safety compliance, patient safety, or workplace safety. We also examined the significance of the direct and indirect effects of organizational support for patient safety and workplace safety on the two safety outcomes through patient safety compliance and workplace safety compliance. For this analysis, we employed bootstrapping with 10,000 bootstrap samples and calculated 95% bias‐corrected confidence intervals (CI), as recommended by Preacher and Hayes (2008).

### 4.4 Ethical considerations

Participation in the original survey was voluntary and anonymous, and participants were informed of their option to withdraw at any point without negative consequences. The study's design and protocols followed the principles delineated in the Declaration of Helsinki, and the study received ethics approval from the Yonsei University Health System Institutional Review Board (4–2023‐1480).

## 5 RESULTS

### 5.1 Sample characteristics

Of the 34 hospitals that participated in the study, about 65% were situated in metropolitan areas, and most were private hospitals (*n* = 28, 82.4%). With regard to nurses' demographics, most were female (*n* = 1180, 94.0%), and the large majority held permanent positions (*n* = 1251, 99.7%). Participants had an average age of 31.2 years and a mean nursing experience of 7.5 years. On average, they had worked 6.7 years in their current hospital and 4.6 years in their current unit. Approximately 58% of nurses (*n* = 730) were working in either medical, surgical, or combined medical‐surgical units (Table 1).

**TABLE 1:** Sample demographic characteristics (*N* = 1255).

	Characteristic	Category	*n* (%) or *M* (SD)
Hospitals (*N* = 34)	Location	Metropolitan area	15 (44.1)
		Non‐metropolitan area	19 (55.9)
	Hospital type	Public	6 (17.6)
		Private	28 (82.4)
	Hospital status	Advanced general	16 (47.1)
		General	18 (52.9)
	Number of beds	900 or above	6 (17.6)
		700–899	14 (41.2)
		500–699	6 (17.6)
		300–499	8 (23.5)
Nurses (*N* = 1255)	Gender	Female	1180 (94.0)
		Male	75 (6.0)
	Age		31.2 (6.3)
	Nursing experience (years)		7.5 (6.3)
	Hospital tenure (years)		6.7 (6.0)
	Unit tenure (years)		4.6 (4.3)
	Employment status	Permanent	1251 (99.7)
		Temporary	4 (0.3)
	Work unit	Medical, surgical or combined medical‐surgical	730 (58.2)
		Specialty	525 (41.8)
	Educational level	Diploma	86 (6.9)
		BSN or higher	1169 (93.1)

*Note*: Specialty units included emergency, critical care, and perioperative units. Abbreviations: *M*, mean; *SD*, standard deviation; BSN, Bachelor of Science in Nursing.

### 5.2 Preliminary analyses

Variance inflation factors between predictors and mediators were within the range of 2.00 to 2.12, indicating no potential problems with multicollinearity (Hair et al., 2010). Skewness and kurtosis values of study variables varied from −0.40 to 0.90 and did not exceed an absolute value of 2 (Curran et al., 1996), suggesting a non‐significant deviation from normality. Table 2 presents Pearson's correlation coefficients and descriptive statistics for key study variables. The correlation coefficients ranged from .35 to .73, indicating varying levels of positive associations among the variables. Although strong associations were observed between organizational support for patient safety and organizational support for workplace safety (*r* = .70, *p* < .001) and between patient safety and workplace safety (*r* = .73, *p* < .001), discriminant validity remained viable. This assertion is supported by findings of a simulation study by Voorhees et al. (2016), which suggested that correlations lower than .75 do not typically pose discriminant validity issues.

**TABLE 2:** Correlations and descriptive statistics for key study variables (*N* = 1255).

Variable	1	2	3	4	5	6
1. Organizational support for patient safety	–					
2. Organizational support for workplace safety	0.70***	–				
3. Patient safety compliance	0.46***	0.43***	–			
4. Workplace safety compliance	0.43***	0.47***	0.68***	–		
5. Patient safety	0.44***	0.43***	0.40***	0.40***	–	
6. Workplace safety	0.44***	0.51***	0.35***	0.40***	0.73***	–
Mean	3.46	3.32	3.86	3.78	2.60	2.47
Standard deviation	0.70	0.73	0.59	0.62	0.65	0.68

*** *p* < .001.

### 5.3 Hypothesis testing

CFA was conducted to evaluate the adequacy of the six‐factor measurement model and determine the extent to which the items were associated with their corresponding latent variables. The measurement model demonstrated a good fit to the data, as evidenced by the following index values: *χ* ^2^ (118) = 822.047, CFI = 0.953, TLI = 0.938, RMSEA = 0.069 and SRMR = 0.059. Standardized loadings, representing the relationships between indicators and their respective latent factors, ranged from 0.52 to 0.90 and were all statistically significant (*p* < .001).

Next, we assessed discriminant validity. In the alternative model, the items related to organizational support for patient safety and organizational support for workplace safety were combined into one variable, while the items related to patient safety compliance and workplace safety compliance were combined into another single variable. The index values for this alternative model showed a slightly poorer fit than those for the original model, with *χ* ^2^ (127) = 1500.936, CFI = 0.907, TLI = 0.888, RMSEA = 0.093, and SRMR = 0.065. Akaike information criterion (AIC) values were used to compare the fit of these non‐nested models (Burnham & Anderson, 2002), and the value for the alternative model (AIC = 39,717.079) was higher than that for the original model (AIC = 39,056.190), indicating poorer fit.

Our hypothesized partial mediation model showed a good fit to the data according to index values: *χ* ^2^ (182) = 925.350, CFI = 0.950, TLI = 0.938, RMSEA = 0.057 and SRMR = 0.061. To determine the better‐fitting model, we removed the direct paths from organizational support for patient safety and organizational support for workplace safety to patient safety and workplace safety in order to specify a full mediation model. Index values for this model also showed reasonable fit: *χ* ^2^ (186) = 1123.618, CFI = 0.937, TLI = 0.924, RMSEA = 0.063, and SRMR = 0.079. However, the original partial mediation model showed a significantly better fit than the alternative full mediation model in a chi‐square difference test (∆*χ* ^2^ = 198.268, ∆ df = 4, *p* < .05).

**FIGURE 2:**
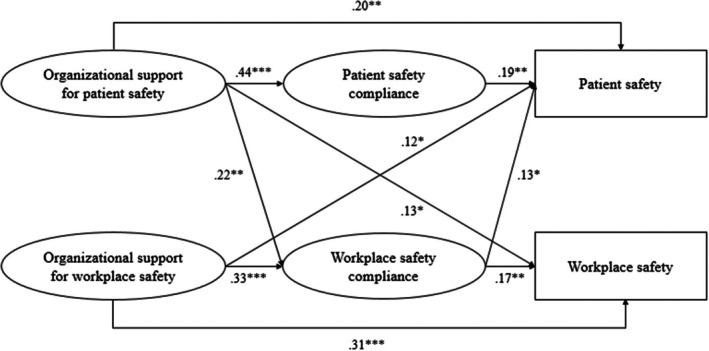
Structural equation modelling (partial mediation) results with standardized coefficient estimates. Nonsignificant paths are not displayed for parsimony. *p < .05, **p < .01, ***p < .001.

As shown in Figure 2, organizational support for patient safety significantly affected both patient safety (*β* = .20, *p* = .002) and workplace safety (*β* = .13, *p* = .048). Similarly, organizational support for workplace safety improved not only patient safety (*β* = .12, *p* = .050) but also workplace safety (*β* = .31, *p* < .001). In addition, organizational support for patient safety was positively associated with patient safety compliance (*β* = .44, *p* < .001), which in turn had a positive effect on patient safety (*β* = .19, *p* = .002). Organizational support for workplace safety was positively related to workplace safety compliance (*β* = .33, *p* < .001), which in turn had a positive impact on workplace safety (*β* = .17, *p* = .006). We also found that patient safety was positively influenced by all the independent and mediator variables, whereas workplace safety was positively influenced by all the variables except patient safety compliance (*β* = .06, *p* = .320).

The distribution of serial indirect effects is skewed in most cases (Preacher & Hayes, 2008). Therefore, we bootstrapped 95% bias‐corrected CIs based on 10,000 samples from the original data. As displayed in Table 3, we found that three of the eight hypothesized indirect paths were statistically significant. Specifically, organizational support for patient safety significantly increased patient safety through patient safety compliance (*β* = .08, 95% CI [0.025, 0.143]). Organizational support for workplace safety significantly enhanced workplace safety through workplace safety compliance (*β* = .06, CI [0.009, 0.102]). Furthermore, organizational support for patient safety had a significant spillover effect on workplace safety via workplace safety compliance (*β* = .04, CI [0.001, 0.072]).

**TABLE 3:** Standardized coefficients of all indirect effects in the hypothesized model (*N* = 1255).

Path	*β*	*p*	95% CI [lower, upper]
Organizational support for patient safety ➔ Patient safety compliance ➔ Patient safety	.08	.006	[0.025, 0.143]
Organizational support for patient safety ➔ Workplace safety compliance➔ Patient safety	.03	.091	[−0.004, 0.060]
Organizational support for patient safety ➔ Patient safety compliance ➔ Workplace safety	.03	.357	[−0.028, 0.078]
Organizational support for patient safety➔ Workplace safety compliance ➔ Workplace safety	.04	.044	[0.001, 0.072]
Organizational support for workplace safety ➔ Patient safety compliance ➔ Patient safety	.02	.334	[−0.016, 0.046]
Organizational support for workplace safety ➔ Workplace safety compliance ➔ Patient safety	.04	.063	[−0.002, 0.087]
Organizational support for workplace safety ➔ Patient safety compliance ➔ Workplace safety	.01	.558	[−0.011, 0.020]
Organizational support for workplace safety ➔ Workplace safety compliance ➔ Workplace safety	.06	.021	[0.009, 0.102]

Abbreviations: CI, bias‐corrected confidence interval.

## 6 DISCUSSION

This study examined whether organizational support for patient safety and workplace safety could improve nurses' patient and workplace safety compliance. In addition, we investigated whether nurses' compliance in these two areas could improve outcomes in patient and workplace safety. Furthermore, we examined the indirect effects of organizational support on patient and workplace safety through nurses' safety compliance. Not only did this study demonstrate the spillover effects of organizational support for patient safety and workplace safety on nurses' safety compliance and subsequent safety outcomes but it also revealed that these relationships are mutually beneficial.

As expected, we found that organizational support for patient safety directly affects both patient and workplace safety outcomes, as does organizational support for workplace safety, supporting H1 and H2. The study findings also show that efforts by organizations to support patient safety have the anticipated effect of improving patient safety and, by extension, workplace safety. These results show the direct spillover effects of organizational support for workplace safety on patient safety. On the whole, the findings indicate that organizational efforts to support patient and workplace safety not only impact patient safety and workplace safety but can yield positive spillover effects on each other, with organizational support for patient safety enhanced workplace safety and organizational support for workplace safety improved patient safety. These findings are consistent with numerous prior studies suggesting a positive association between organizational support for safety and safety outcomes in a single domain (e.g., Moda et al., 2021) and add concrete evidence for spillover effects across safety domains (e.g., Sturm et al., 2019).

In addition, nurses with a greater perception of organizational support for patient safety reported higher compliance with both patient and workplace safety. However, nurses with a greater perception of organizational support for workplace safety reported higher compliance only with workplace safety. These findings suggest that an organization's emphasis on patient safety has a spillover effect on nurses' safety behaviour, enhancing their compliance with workplace safety as well as patient safety, but that the positive effect of support for workplace safety is limited to nurses' workplace safety compliance. Similarly, nurses' patient safety compliance improved only patient safety, whereas their workplace safety compliance positively affected both patient safety and workplace safety. Although these findings support our H3, H4, H5 and H6, they also indicate that spillover effects between the patient safety and workplace safety domains may differ depending on the specific outcome under investigation, underscoring a need for further research in this area.

With regard to the mediating roles of nurses' safety compliance, organizational support for patient safety positively affected patient and workplace safety through patient safety compliance and workplace safety compliance, respectively. However, when organizational support for workplace safety was the independent variable, the indirect effect was significant only when workplace safety was increased by workplace safety compliance. In the mediation analyses, three of the eight mediation paths were found to be significant, indicating that H7 was partially supported. Although our mediation analyses supported the existing belief that organizational enabling factors such as organizational support promote worker safety behaviours that lead to improved safety outcomes (Bisbey et al., 2021), this serial mediation was significant in a limited number of paths. This result may be largely due to the non‐significant paths from organizational support for workplace safety to patient safety compliance and from patient safety compliance to workplace safety. The non‐significant mediating effects observed in this study should be re‐evaluated in future research.

Our findings highlight the significant mutual benefits of promoting both patient safety and workplace safety in the management of healthcare organizations. From a theoretical perspective, our findings provided convincing evidence for the assumption that a connection exists between protecting the safety of patients and protecting the safety of workers—in other words, that a synergy exists between the two. Previous researchers have argued that workplace safety and patient safety are equally important and inseparable (e.g., Spratt et al., 2012). Earlier studies have suggested that interventions to improve workplace safety have also led to improved patient safety outcomes (Dutra & Guirardello, 2021; McFadden et al., 2015) and that the perception of inadequate workplace safety among hospital staff has been associated with poorer patient outcomes (Sturm et al., 2019). However, no previous research known to us has examined the interplay between organizational support for patient safety and organizational support for workplace safety and shown their joint impact on safety outcomes in the healthcare context. Additionally, our findings provide empirical support for Flin's (Flin, 2007) theoretical model in showing that patient safety and workplace safety are not separate issues but rather are mutually reinforcing.

Our study underscores the importance of promoting and managing safety for both patients and workers in healthcare organizations. Based on our results, healthcare organizations should strategically coordinate and implement policies and strategies that simultaneously prioritize patient safety and workplace safety. One recommended approach is to regularly convene interprofessional committees to propose, implement, and oversee systematic methods for mitigating patient and workplace safety issues (Bromley & Painter, 2019). Additionally, conducting regular safety climate assessments at the unit level could offer insights into staff perceptions of safety issues (Hofmann et al., 2017). Furthermore, promoting open communication through empowering leadership has been found to be more effective than coercion in promoting adherence to safe work practices (Aljabri et al., 2020). Another helpful, practical strategy is safety training. As Labrague et al. (2021) asserted, training programs involving both supervisors and staff are essential for enhancing communication and understanding of workplace safety protocols and safety responsibilities within units. Specific training for recognizing and comprehending risks and common injuries in each work area can also diminish the likelihood of safety issues (Aljabri et al., 2020). Lastly, providing sufficient physical (e.g., PPE) and psychosocial resources (e.g., safety procedures and social support) to nurses are also necessary (Delgado et al., 2020).

### 6.1 Limitations

To the best of our knowledge, this is the first to provide empirical evidence of the spillover effects between patient safety and workplace safety in healthcare settings. Furthermore, it substantiates the trickle‐down impact of organizational support for safety on nurses' compliance behaviour and safety outcomes in the healthcare context, laying a solid groundwork for subsequent studies in this area. Nonetheless, our study is limited by its sole reliance on survey data collection and the use of cross‐sectional data. The reliance on self‐reported perceptions from nurses regarding their organizations' support for safety and their own safety compliance may introduce common method bias. While our analysis was guided by a theoretical framework suggested by Flin (2007), the cross‐sectional nature of the study and the data drawn from a single survey restrict our ability to deduce causal relationships. Although collecting data from a broad spectrum of hospitals in South Korea stands as a strength of this study, its confinement to the Korean context may hinder the global applicability of its findings. Future studies should seek to broaden the examination of patient safety and workplace safety through more sophisticated methodologies. While CFA was employed to validate our findings, the robustness of future research would benefit from incorporating objective measures of actual accidents, incidents, and injuries (Gracia et al., 2020).

## 7 CONCLUSIONS

To the best of our knowledge, no prior research has explored the simultaneous impact of organizational support for patient safety and organizational support for workplace safety and their spillover effects on safety outcomes in healthcare settings. Consequently, this study provides new and significant insights into the intertwined nature of patient safety and workplace safety in this context. Our findings reveal that organizational support for both patient safety and workplace safety directly impacts the respective domains and can produce positive spillover effects—that is, organizational efforts to improve patient safety can have beneficial consequences for workplace safety, and organizational support for workplace safety can enhance patient safety. Additionally, our findings include significant indirect paths between organizational support for safety and patient and workplace safety outcomes. Overall, the study highlights the importance of integrating patient and workplace safety strategies in healthcare settings, reinforcing the need for a holistic approach to achieve better safety outcomes.

## AUTHOR CONTRIBUTIONS

JKS and SEL contributed equally to this work. They collaboratively designed the study, developed the methodology and performed the analysis. Each author was involved in drafting and revising the manuscript, and both have approved the final version for publication.

## FUNDING INFORMATION

This study was supported by a National Research Foundation of Korea grant funded by the Korean Government (MSIT) (No. RS‐2023‐00208138).

## CONFLICT OF INTEREST STATEMENT

No conflict of interest has been declared by the authors.

## PEER REVIEW

The peer review history for this article is available at https://www.webofscience.com/api/gateway/wos/peer‐review/10.1111/jan.16222.

## Supporting information


Data S1:

## Data Availability

The data that support the findings of this study are available on request from the corresponding author. The data are not publicly available due to privacy or ethical restrictions.
